# A new combination expands the range of the African araneid spider *Singafrotypa* (Araneae, Araneidae)

**DOI:** 10.3897/zookeys.207.3522

**Published:** 2012-07-11

**Authors:** Anna Šestáková, Mikhail M. Omelko

**Affiliations:** 1 Zoological Museum, University of Turku, FI-20014, Turku, Finland; 2 Department of Zoology, Faculty of Natural Sciences, Comenius University, Mlynská dolina, 84215 Bratislava, Slovakia; 3 Far Eastern Federal University, Sukhanova, 8, Vladivostok 690950 Russia; 4 Gornotaezhnaya Station FEB RAS, Gornotaezhnoe Vil.,Ussuriyski Dist., Primorski Krai 692533 Russia

**Keywords:** *Larinioides subinermis*, *Singafrotypa*, redescription, taxonomy, spider, Africa

## Abstract

Study of the syntype of *Larinioides subinermis*, a species known from Ethiopia only, revealed that it actually belongs to *Singafrotypa* Benoit, 1962. We redescribe *Singafrotypa subinermis* (Caporiacco, 1940), **comb. n.**, and provide a key to females of four species belonging to *Singafrotypa*. A distribution map for all species is provided.

## Introduction

The small African orb-weaver genus *Singafrotypa* was found to be restricted to western, southern and central Africa (Fig. 14). It presently contains 3 species: *Singafrotypa acanthopus* (Simon, 1907), *Singafrotypa mandela* Kuntner & Hormiga, 2002 and *Singafrotypa okavango* Kuntner & Hormiga, 2002 (Kuntner and Hormiga 2002). Examination of syntypes of *Larinioides subinermis* revealed its generic affinity to *Singafrotypa* and therefore expands the known diversity of this genus. Except for the original description based on a female, it was considered in two further taxonomic publications by Grasshoff (1970, 1983) who examined the types. Grasshoff (1970) indicated that *Larinioides subinermis* belonged to Cyclosini, although *Larinioides* is a member of Araneini (Grasshoff 1983) he did not make any formal transfer. When Grasshoff returned the types to MZUF he noted that the species actually belonged to *Singafrotypa* Benoit, 1962 and considered it as a junior synonym of *Singafrotypa acanthopus* (Simon, 1907) (Berdondini and Whitman 2002).

Our study of a syntype of *Larinioides subinermis* showed that Grasshoff’s informal synonymy was not correct. This became evident after studying the recently published revision of *Singafrotypa* by Kuntner and Hormiga (2002). Although *Larinioides subinermis* is rather similar to *Singafrotypa acanthopus*, the type species of the genus, it has clear differences.

In this paper we redescribe *Larinioides subinermis* and propose a new combination as *Singafrotypa subinermis* (= *Larinioides subinermis*), comb.n.

## Material and methods.

Photographs were made with an Olympus Camedia E-520 camera attached to an Olympus SZX16 stereomicroscope at the Zoological Museum, University of Turku. Digital images were montaged using “CombineZP” image stacking software. Examined material is deposited in Museo Zoologico “La Specola” dell’Universita di Firenze, Florence, Italy (MZUF). The terminology of epigynal morphology follows Kuntner & Hormiga (2002). All measurements are in millimetres.

Abbreviations: BL – basal lamella of epigyne; CO – copulatory openings; EB – epigynal base; LL – lateral lamella of epigyne; MP – median plate of epigyne; SC – scapus.

## Taxonomy

### *Singafrotypa* Benoit, 1962

#### Singafrotypa subinermis

(Di Caporiacco, 1940) comb. n.

http://species-id.net/wiki/Singafrotypa_subinermis

Figs 1–5 , 9, 13

Larinioides subinermis Di Caporiacco 1940: 821, f. 28 (♀).

##### Material.

1♀ Syntype, Coll N°72, Mag. N°2581, Ethiopia, Lago Regina Margherita on island, 16.1,1938 (L. Di Capporiacco)

**Diagnosis.** *Singafrotypa subinermis* can be recognized from other females of *Singafrotypa* by the relative proportion of the scapus to the epigynal base (ventral view) – tip of the scapus only slightly protruding over the base of the epigyne, and position of copulatory openings on the edge of the epigynal base (Figs 6–9). Unlike *Singafrotypa okavango*, it does not have a heart-shaped epigynal base and a long, distinctly wrinkled scapus (Figs 8, 12). It differs from *Singafrotypa mandela* by the absence of stout macrosetae on the palpal tarsus and paturon, a conical palpal tarsus (Kuntner and Hormiga 2002), and in the shape of the epigynal base (Figs 7, 11). The epigyne of *Singafrotypa subinermis* is the most similar to *Singafrotypa acanthopus* (Figs 6, 10), but it differs from latter by having fewer wrinkles on the scapus with a round tip (triangular in *Singafrotypa acanthopus*), and the shallow depression of the median plate without protruding lateral lamellae (Figs 9, 13).

##### Description.

**Figures 1–5.**
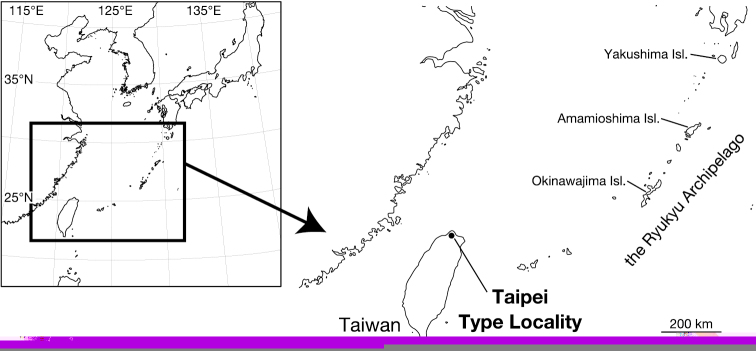
Female of *Singafrotypa subinermis*. **1** ventral **2** dorsal **3** pedipalp, retrolateral **4** epigyne, dorsal **5** ibid., posterior.

Female. Total length 11.6. Carapace 4.2 long, 3.2 wide. Length of patella + tibia I 3.8. Carapace uniform red-brown, covered with small white hairs; cephalon protruding. Diameter of AME is 1.3 times larger than PME. Distance between AME 2 times longer than between PME. Chelicerae dark brown; 4 promarginal teeth, 3 retromarginal teeth. Sternum, brown, anteriorly in the middle with short, indistinct pale stripe; longer than wide (Fig. 1). Abdomen elongated, yellowish with two longitudinal brown stripes (Fig. 2), ventrally yellow, paler between epigastric furrow and spinnerets (Fig. 1). Legs yellow. Palp normal, no conical tarsus (Fig. 3). Femur I with 1 prolateral spine; 3 small, dorsal spines; no retrolateral spines.

Epigyne as in Figs 4, 5, 9, 13. Epigyne well sclerotized, protruding, with well developed scapus; epigynal base as wide as long, narrowing anteriorly (dorsal view); basal lamella thin, poorly developed; median plate with shallow depression (under scapus); copulatory openings located anteriorly on the edge of the base; flexible scapus almost as long as epigynal base, indistinctly wrinkled with a round tip (Figs 4, 9).

**Figures 6–13.**
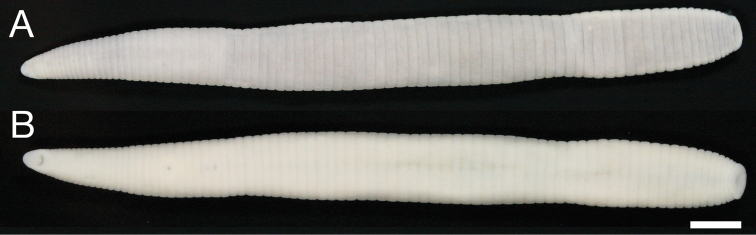
Epigynes of *Singafrotypa*. **6, 10** *Singafrotypa acanthopus* **7, 11** *Singafrotypa mandela* **8, 12** *Singafrotypa okavango* **9, 13** *Singafrotypa subinermis* **6–9** epigyne, dorsal **7–13**, ibid., posterior (Figs **6–8, 7–12** redrawn with permission, after Kuntner and Hormiga 2002).

##### Distribution.

**Figure 14.**
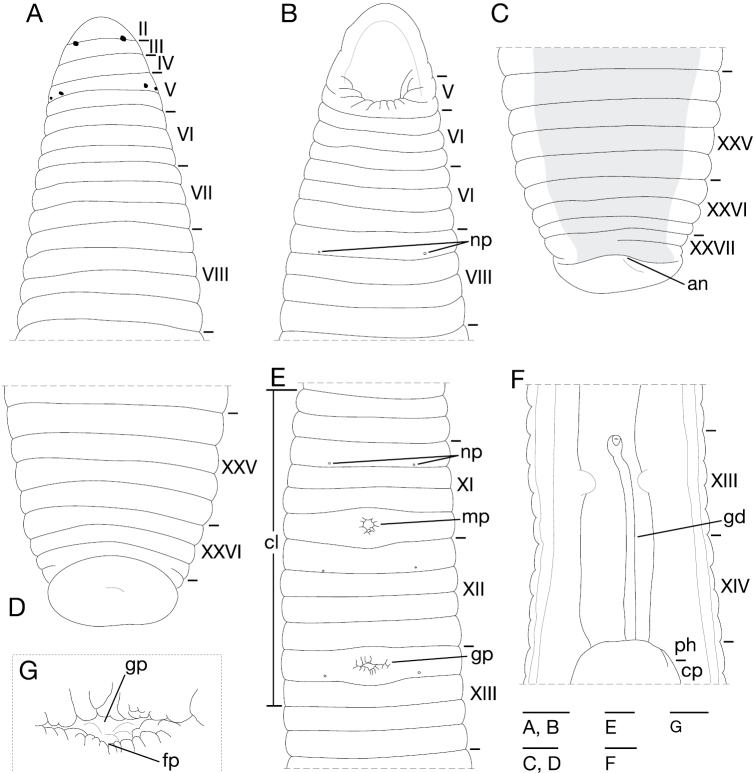
Distribution of the species of *Singafrotypa* (after Kuntner & Hormiga 2002 with additional locality of *Singafrotypa subinermis*). (http://upload.wikimedia.org/wikipedia/en/2/21/Africa_satellite_orthographic.jpg ) ♦ *Singafrotypa acanthopus* ■ *Singafrotypa mandela* ● *Singafrotypa okavango* ★ *Singafrotypa subinermis*.

Only known from the type locality, islands of Lake Abaya in Ethiopia (Fig. 14). *Singafrotypa subinermis* is the easternmost species of the genus.

### Key for females of *Singafrotypa*

**Table:** 

1	Epigynal base (ventral) oval or round	2
–	Epigynal base heart-shaped; long wrinkled scapus (Figs 8, 12)	*Singafrotypa okavango*
2	Copulatory openings in the middle or more anteriorly on epigynal base (ventral); epigynal base (ventral) as wide as long; palpal tarsus not conical; chelicerae and palpal tarsus without stout macrosetae	3
–	Copulatory openings posteriorly on epigynal base; epigynal base wider than long (Figs 7, 11); stout short macrosetae on palpal tarsus, and laterally on paturon	*Singafrotypa mandela*
3	Scapus with many wrinkles, protrudes over epigynal base (ventral); copulatory openings in the middle of epigynal base (ventral); deep depression of median plate anteriorly with protruding lateral lamellae (posterior) (Figs 6, 10)	*Singafrotypa acanthopus*
–	Scapus with few wrinkles, does not protrude over epigynal base; copulatory openings anteriorly on epigynal base (ventral); shallow depression of median plate without protruding lateral lamellae (posterior) (Figs 9, 13)	*Singafrotypa subinermis*

## Supplementary Material

XML Treatment for Singafrotypa subinermis
